# Evaluating the generalizability of deep learning image classification algorithms to detect middle ear disease using otoscopy

**DOI:** 10.1038/s41598-023-31921-0

**Published:** 2023-04-01

**Authors:** Al-Rahim Habib, Yixi Xu, Kris Bock, Shrestha Mohanty, Tina Sederholm, William B. Weeks, Rahul Dodhia, Juan Lavista Ferres, Chris Perry, Raymond Sacks, Narinder Singh

**Affiliations:** 1 Faculty of Medicine and Health, University of Sydney, Sydney, NSW Australia; 2 Department of Otolaryngology, Head and Neck Surgery, Westmead Hospital, Sydney, NSW Australia; 3 AI for Good Lab, Microsoft, Redmond, WA USA; 4 Azure FastTrack Engineering, Brisbane, QLD Australia; 5 University of Queensland Medical School, Brisbane, QLD Australia; 6 Microsoft, Redmond, WA USA

**Keywords:** Diagnosis, Public health, Translational research

## Abstract

To evaluate the generalizability of artificial intelligence (AI) algorithms that use deep learning methods to identify middle ear disease from otoscopic images, between internal to external performance. 1842 otoscopic images were collected from three independent sources: (a) Van, Turkey, (b) Santiago, Chile, and (c) Ohio, USA. Diagnostic categories consisted of (i) normal or (ii) abnormal. Deep learning methods were used to develop models to evaluate internal and external performance, using area under the curve (AUC) estimates. A pooled assessment was performed by combining all cohorts together with fivefold cross validation. AI-otoscopy algorithms achieved high internal performance (mean AUC: 0.95, 95%CI: 0.80–1.00). However, performance was reduced when tested on external otoscopic images not used for training (mean AUC: 0.76, 95%CI: 0.61–0.91). Overall, external performance was significantly lower than internal performance (mean difference in AUC: −0.19, p ≤ 0.04). Combining cohorts achieved a substantial pooled performance (AUC: 0.96, standard error: 0.01). Internally applied algorithms for otoscopy performed well to identify middle ear disease from otoscopy images. However, external performance was reduced when applied to new test cohorts. Further efforts are required to explore data augmentation and pre-processing techniques that might improve external performance and develop a robust, generalizable algorithm for real-world clinical applications.

## Introduction

It estimated that over 1.5 billion people in the world live with hearing loss, representing nearly 20% of the global population^1^. The prevalence of hearing loss is expected to rise to over 2.5 billion by 2050^1^. In the US, it is estimated that the overall cost of deafness and hearing loss amounts to US$980 billion annually^2^. Direct and indirect costs related to hearing loss are comprised of health sector costs, educational support, loss of productivity, and societal costs^3,4^. In the global pediatric population, 34 million children experience hearing loss, of which, 60% can be attributed to preventable causes^5^. The WHO estimates that over one-third of preventable childhood hearing loss is attributed to infections including mumps, rubella, meningitis, measles and middle ear infections^5^.

Otoscopy is an important component of ear health and hearing assessments to identify ear infections. Otoscopy is performed routinely by multiple healthcare workers including medical students, nurses, general practitioners, emergency medicine physicians, paediatricians, audiologists, and otolaryngologists. However, the ability to accurately interpret otoscopic findings varies by user experience^6–10^. Pichichero et al. (2005) demonstrated differences between general practitioners, paediatricians, and otolaryngologists to recognise tympanic membrane (TM) abnormalities^8^. Significant performance differences were found when non-otolaryngologists were asked to differentiate between acute otitis media (AOM), otitis media with effusion (OME), or retracted TMs^8^. These findings are consistent with recent studies that demonstrate that diagnostic accuracy is reduced when non-experts must differentiate between multiple ear disease sub-types^6,11^. Otolaryngologists significantly outperformed non-otolaryngologists to identify ear disease sub-types and important pathological characteristics (e.g. tympanic membrane perforations, attic retraction, or myringitis)^6,8^.

Accurately recognising middle ear diseases is important for early management, prevention of long-term hearing loss, can improve speech and language development and reduce healthcare costs^12^. However, the subjective and often inconsistent nature of traditional otoscopy makes accurate interpretation difficult. To address this challenge, deep learning -based image classification algorithms have been developed using machine learning techniques^13–35^. Traditional machine learning algorithms rely on statistical models and pre-defined features, whereas deep learning algorithms use neural networks to learn features and extract patterns directly from raw data^13,36,37^. Deep learning methods have the potential to improve early ear disease detection by bridging the gap between expert and non-expert performance and enhancing the performance of previously developed machine learning models for otoscopy^38^.

Previous studies have explored image classification algorithms for otoscopy using deep learning approaches^13–35^. In a recent systematic review and meta-analysis, a pooled analysis of 14 studies achieved 91% accuracy in differentiating between normal versus abnormal otoscopic images^39^. From a pooled analysis of three studies, multi-classification algorithms achieved 98% to differentiate AOM, OME, and normal aerated ears without middle ear pathology^39^. Despite high levels of accuracy reported, the binary and multi-classification analyses yielded substantial analyses heterogeneity between studies^39^. Variability between models to classify ear disease subtypes suggests performance may not be generalisable to new test environments.

The inability to generalize to environments beyond those used for training could limit the clinical utility and sustainability of deep learning algorithms for otoscopy. Variability in data source, capture devices, ear disease sub-type, and deep learning methods may impact classification performance in clinical encounters different to those used for training. To date, an exploration of the generalisability of deep learning algorithms for otoscopy has not been evaluated.

Deep learning techniques have been shown to excel in tasks such as image classification, with the ability to classify images with high accuracy and to generalize to new data^40,41^. Unlike traditional machine learning techniques, deep learning algorithms can automatically extract relevant features from images, reducing the need for manual feature engineering. This makes deep learning algorithms less sensitive to variations in data quality and pre-processing steps, and more robust to changes in data source, capture devices, and ear disease subtypes.

The purpose of this study was to construct a deep learning-based image classifier capable of differentiating between normal and abnormal otoscopic images using three independently collected otoscopy databases, evaluate the generalisability of the algorithm on images not used for training, and explore the optimal convolutional neural network (CNN) and deep learning approach to optimise performance.

## Methods

### Ethics

This research study was conducted in accordance with the Helsinki Declaration, the Standard for Reporting Diagnostic Accuracy Studies (STARD) and the Consolidated Standards of Reporting Trials for interventions involving artificial intelligence (CONSORT-AI) reporting guidelines^42,43^. This study utilised three open-access otoscopic image databases that were collected after obtaining Institutional Review Board (IRB) approval from each of the respective human research ethics committees and published online for public access, as indicated by each of the authorship groups (Bitlis Eren University Ethics Committee^34,44^, University of Chile Scientific and Research Ethics Committee^18^, Ohio State University Institutional Review Board^45^).

### Data sources

Otoscopic images ascertained for the purposes of this study can be found elsewhere for online public access, as per the authorship groups^18,34,45^. In Turkey (Cohort A), 848 otoscopic images were collected at the Özel Van Akdamar Hospital using digital video otoscopes (unspecified brand and model) with ground-truth classified by two otolaryngologists and one paediatrician as: normal, acute otitis media (AOM), and chronic otitis media (COM) consisting of chronic suppurative otitis media^34^. In Chile (Cohort B), 540 otoscopic images were collected at the University of Chile Clinical Hospital using the Firefly DE500 digital video otoscope (Firefly Global, Belmont, MA, US. 2022) with ground-truth classified by 1 otolaryngologist as: normal or COM^18^. In the United States (Cohort C), 454 otoscopic images were collected at Nationwide Children’s Hospital and Ohio State University using the Jedmed Horus+ (Jedmed, St Louis, MO, US, 2022) with ground-truth classified by 1 otolaryngologist as: normal or otitis media with effusion (OME)^45^. Otoscopic images where the TM could not be visualised were excluded (e.g., obstruction with wax or foreign body).

### Algorithm development

Deep learning-based binary class algorithms were developed to classify normal or abnormal otoscopic images using Cohort A, B and C independently. Abnormal sub-classes included AOM, OME, and COM. Pre-existing ground-truth labels were available for each otoscopic image from the online data sources^18,34,45^. We used four different neural net architectures including ResNet-50 (comprised of 50 layers and 23 million parameters)^46^, VGGNet-16 (comprised of 16 layers and 134 million parameters)^47^, DenseNet-161 (comprised of 16 layers and 26 million parameters)^48^, and Vision Transformer (comprised of 12 layers and 85 million parameters)^49^. Images were resized to 224 by 224 pixels. Each model was trained for 200 epochs using the Stochastic Gradient Descent (SGD) with a learning rate = 0.003 as the optimizer. The loss function is cross entropy. Training was completed using the Microsoft Azure Machine Learning Studio (Redmond, Washington, USA). All the models used the weights of models trained on the ImageNet dataset as their initial weights. Raw data was used for the purposes of developing the model and validation.

To assess the generalizability of a model trained on a single-source dataset, we first randomly split the dataset from one single source into training and validation and testing sets, allocating 70% for training, 10% for validation and 20% for testing (internal validation). We trained the model using the training set and selected the final model as the one at the best-performing epoch minimizing the loss on the validation set. Next, we tested the final model on the testing (internal validation) set. Then, we tested the final model on the two external validation cohorts. To ensure the robustness and reliability of the results, we ran each experiment for three times, and the mean and standard deviation of accuracy, area under the curve (AUC), sensitivity, and specificity were computed, as shown in Table 3. This was done to account for any potential variability or instability in the results due to the stochastic nature of deep learning algorithms. The mean and standard deviation values provided a more comprehensive understanding of the performance of the models, as well as the variability of their performance across multiple runs.

To evaluate the pooled performance, we first combined all the data from the three cohorts, and then used fivefold cross-validation as follows: (1) The data was randomly split into fivefolds; (2) a model was trained using the training set consisting of three of the five folds. We selected the final model as the one at the best-performing epoch minimizing the loss on the validation set consisting of one of the five folds. We tested the final model on the remaining fold. (3) Step 2 was repeated five times until each fold had been used as a test fold exactly once. All the metrics were averaged to estimate the pooled performance.

### Statistical analysis

**Figure 1:**
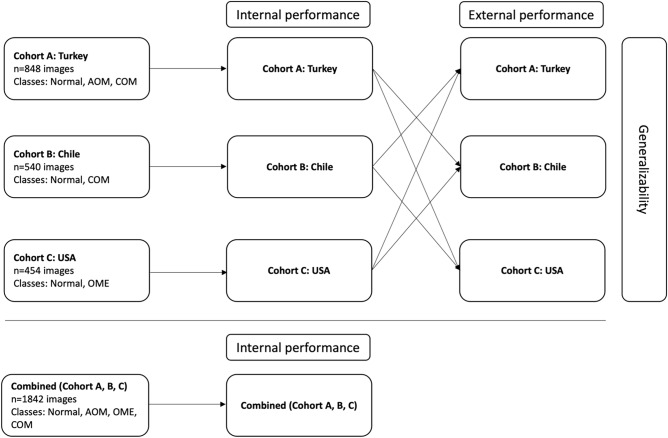
Flow chart of proposed study design.

To compare the algorithm to the reference standard for Cohorts A, B, and C, contingency tables were generated to summarise Area Under the Curve (AUC), accuracy, sensitivity, and specificity. To evaluate internal performance, models were trained and tested using otoscopic images from the same cohort (e.g., split Cohort A otoscopic images into training and test sets). To evaluate external validation performance and generalizability, models were validated on external cohorts (e.g., trained using Cohort A and validated on Cohort B and Cohort C, Fig. 1)^50^. Mean difference in AUC were calculated to compare internal and external performance. We used bootstrapping to compare the Receiver Operating Characteristics (ROC) curves, and the null hypothesis is that the external validation AUC is greater or equal to the internal validation AUC^50^. Pooled assessment was performed by combining Cohort A, B, and C and fivefold cross-validation was applied (Fig. 1).

## Results

In total, 1842 otoscopic images were collected from three online resources, comprised of 1117 normal and 725 abnormal images. Table 1 summarises the distribution of normal and abnormal otoscopic images by Cohort A, B and C.

**Table 1:** Summary of data sources.

	Otoscope	Image resolution (pixels)	Ground-truth	Total images	Normal images	Abnormal images	Ear disease diagnoses	Published accuracy
Cohort A (Özel Van Akdamar Hospital, Van, Turkey)	Unspecified brand/model	500 × 500 (DPI: 72)	2 otolaryngologists, 1 paediatrician	848	578	270	Normal, AOM, COM	99.4% (857 training images including AOM, OME, COM, Wax obstruction)
Cohort B (University of Chile’s Hospital, Santiago, Chile)	Firefly DE500 digital video otoscope	420 × 380 (DPI: 72)	1 otolaryngologist	540	360	180	Normal, COM	93.9%
Cohort C (Nationwide Children’s Hospital, Columbus, Ohio, US)	JEDMED HORUS + digital video otoscope	1397 × 982 to 441 × 355 (DPI: 72)	1 otolaryngologist	454	179	275	Normal, OME	80.6%
Combined (Cohort A, B, C)	–	–	–	1842	1117	725	Normal, AOM, OME, COM	–

*AOM* acute otitis media, *COM* chronic otitis media, *OME* otitis media with effusion, *DPI* dots per inch.

### Internal performance

Table 2 summarises internal performance for each cohort. Sensitivity and specificity results are provided in the Supplementary Appendix. Models trained using Cohort A otoscopic images achieved AUC between 0.82 and 0.86 (accuracy: 80 to 84%; sensitivity: 57 to 70%; specificity 90 to 91%). Models trained using Cohort B otoscopic images achieved an AUC of 1.00 (100% accuracy, 100% sensitivity, and 100% specificity). Models trained using Cohort C otoscopic images achieved AUC between 0.98 to 0.99 (accuracy: 91 to 95%; sensitivity: 91 to 96%; specificity: 89 to 96%).

**Table 2:** Summary of internal and external performance between Cohorts to differentiate normal versus abnormal otoscopic images trained using ResNet-50, DenseNet-161, VGG16, vision transformer.

Experiments		ResNet-50		DenseNet-161		VGG16		Vision Transformer	
Train	Validate	Acc % (SD)	AUC	Acc % (SD)	AUC	Acc % (SD)	AUC	Acc % (SD)	AUC
Cohort A	Cohort A (internal)	78 (0.02)	0.83 (0.01)	83 (0.01)	0.86 (0.01)	82 (0.01)	0.84 (0.0)	82 (0.0)	0.85 (0.02)
	Cohort B (external)	75 (0.05)	0.88 (0.01)	65 (0.04)	0.84 (0.03)	80 (0.03)	0.89 (0.02)	62 (0.06)	0.86 (0.02)
	Cohort C (external)	75 (0.01)	0.81 (0.02)	67 (0.03)	0.71 (0.05)	73 (0.03)	0.78 (0.05)	65 (0.02)	0.64 (0.06)
Cohort B	Cohort B (internal)	100 (0.0)	1.0 (0.0)	100 (0.0)	1.0 (0.0)	100 (0.0)	1.0 (0.0)	100 (0.0)	1.0 (0.0)
	Cohort A (external)	59 (0.04)	0.62 (0.02)	56 (0.07)	0.67 (0.05)	64 (0.02)	0.63 (0.01)	67 (0.03)	0.62 (0.05)
	Cohort C (external)	76 (0.02)	0.87 (0.03)	81 (0.01)	0.89 (0.01)	77 (0.01)	0.9 (0.01)	67 (0.03)	0.82 (0.04)
Cohort C	Cohort C (internal)	92 (0.01)	0.99 (0.0)	92 (0.01)	0.98 (0.0)	94 (0.01)	0.99 (0.0)	96 (0.01)	0.99 (0.0)
	Cohort A (external)	53 (0.12)	0.69 (0.01)	52 (0.04)	0.66 (0.03)	66 (0.01)	0.58 (0.02)	53 (0.11)	0.57 (0.03)
	Cohort B (external)	37 (0.02)	0.86 (0.01)	36 (0.01)	0.92 (0.01)	41 (0.03)	0.92 (0.02)	38 (0.02)	0.9 (0.01)

*Acc* accuracy, *AUC* area under the receiver operating characteristics curve.

Table 2 summarises external validation performance for each cohort. Models trained using Cohort A otoscopic images and tested on Cohort B achieved AUC between 0.80 and 0.87 (accuracy: 62 to 77%; sensitivity: 76 to 96%; specificity: 47 to 78%), while those tested on Cohort C achieved AUC between 0.61 and 0.82 (accuracy: 65 to 76%; sensitivity: 80 to 92%; specificity: 25 to 69%).

Models trained using Cohort B otoscopic images and tested on Cohort A achieved AUC between 0.60 to 0.67 (accuracy: 59 to 65%; sensitivity: 4 to 54%; specificity: 61 to 99%), while those tested on Cohort C achieved AUC between 0.87 and 0.91 (accuracy: 72 to 80%; sensitivity: 62 to 73%; specificity: 88 to 95%) (Table 2).

Models trained using Cohort C otoscopic images and tested on Cohort A achieved AUC: 0.54 to 0.68 (accuracy: 44 to 70%; sensitivity: 14 to 82%; specificity: 26 to 96%), while those tested on Cohort B achieved AUC: 0.85 to 0.94 (accuracy: 34 to 45%; sensitivity: 100%; specificity: 1 to 17%) (Table 2).

On average, external AUC was significantly lower than internal AUC (range of mean difference in AUC for DenseNet-161: 0.07–0.39). As shown in Table 3, Cohort A had the smallest difference between internal and external AUC between architectures (range of mean differences: −0.05 to 0.07).

**Table 3:** Summary of mean difference in area under the curve (AUC) estimates between internal and external performance to differentiate normal versus abnormal otoscopic images trained using DenseNet-161.

	Train	External	Mean difference in AUC (internal – external)	p-value
ResNet-50	Cohort A	Cohort B	−0.05	0.87
	Cohort A	Cohort C	0.01	0.42
	Cohort B	Cohort A	0.40	< 0.01
	Cohort B	Cohort C	0.09	< 0.01
	Cohort C	Cohort A	0.31	< 0.01
	Cohort C	Cohort B	0.14	< 0.01
DenseNet-161	Cohort A	Cohort B	0.07	0.04
	Cohort A	Cohort C	0.14	< 0.01
	Cohort B	Cohort A	0.39	< 0.01
	Cohort B	Cohort C	0.10	< 0.01
	Cohort C	Cohort A	0.36	< 0.01
	Cohort C	Cohort B	0.07	< 0.01
VGG16	Cohort A	Cohort B	−0.03	0.79
	Cohort A	Cohort C	0.12	< 0.01
	Cohort B	Cohort A	0.37	< 0.01
	Cohort B	Cohort C	0.09	< 0.01
	Cohort C	Cohort A	0.43	< 0.01
	Cohort C	Cohort B	0.05	< 0.01
Vision transformer	Cohort A	Cohort B	−0.04	0.85
	Cohort A	Cohort C	0.24	< 0.01
	Cohort B	Cohort A	0.33	< 0.01
	Cohort B	Cohort C	0.13	< 0.01
	Cohort C	Cohort A	0.45	< 0.01
	Cohort C	Cohort B	0.07	< 0.01

### Pooled performance

Models developed using a combination dataset of all cohorts achieved 90 to 91% accuracy (AUC: 0.96; sensitivity: 84 to 87%; specificity: 93 to 95%) (Table 4). DenseNet-161 had the highest AUC and smallest standard deviation for fivefold cross validation.

**Table 4:** Summary of pooled performance to differentiate normal versus abnormal otoscopic images using combined dataset (cohort A, B, and C) by pre-trained CNN architecture.

Model architecture	Acc (SD)	AUC (SD)	Sen (SD)	Spec (SD)
ResNet-50	0.91 (0.02)	0.96 (0.01)	0.87 (0.04)	0.93 (0.01)
DenseNet-161	0.91 (0.02)	0.96 (0.01)	0.86 (0.04)	0.94 (0.01)
VGG16	0.90 (0.02)	0.96 (0.01)	0.84 (0.02)	0.94 (0.02)
Vision transformer	0.92 (0.01)	0.96 (0.01)	0.86 (0.01)	0.95 (0.01)

*Acc* accuracy, *AUC* area under the receiver operating characteristics curve, *Sen* sensitivity, *Spec* specificity, *SD* standard deviation.

## Discussion

Otoscopy is a common clinical task performed by multiple healthcare workers and practitioners with varying levels of expertise. Identifying normal anatomical landmarks and abnormal pathological processes is important in evaluating ear health. The diagnosis of ear disease relies on a combination of presenting symptoms, clinical history, tympanometry, audiometry, and otoscopic findings. AI-based algorithms have been explored in healthcare to augment existing clinical practices in order to enhance judgement and decision-making^51^. AI-based algorithms for otoscopy have achieved substantial accuracy to differentiate between normal or abnormal otoscopic images utilising pre-trained CNN architectures^52–56^. Despite achieving high performance in training and internal validation groups, substantial heterogeneity has been found between model results^39^. The differences may, in part, be attributed to the variability in image capture methods or devices, definition of diagnoses, ground truth determination, sample populations, and methodology used for algorithm development^39^. Translating performance achievements from training environments to new test cohorts is an important aspect for an AI-based model to be generalizable for routine clinical practice. Without achieving generalizable performance and real-world utility^57^, it is unclear if applying AI to otoscopy practices will contribute to improving clinical practice. The purpose of this study was to evaluate the generalisability of a binary classification AI-otoscopy algorithm to classify normal or abnormal otoscopic images, using three independently collected databases and trained using standardised deep learning methods.

This study demonstrates substantial internal performance within each cohort to differentiate between normal or abnormal otoscopic images. Between CNN architectures, models developed using DenseNet-161 achieved the highest internal performance for the pooled assessment. However, when the models were applied externally (i.e., to new validation cohorts not used for training), overall model performance was reduced. The extent of performance variability was dependent on the outcome measure assessed (e.g., accuracy, AUC, sensitivity, and specificity). Performance was reduced in most external comparisons, although the mean difference varied by outcome measure. Cohort A (Özel Van Akdamar Hospital, Van, Turkey) was found to have the smallest external performance drop in accuracy, AUC, and sensitivity. On the other hand, Cohort B (University of Chile’s Hospital, Santiago, Chile) was found to have the smallest performance drop in specificity, but the greatest performance drop in AUC and sensitivity. These findings demonstrate that measuring external model performance and evaluating generalizability to new cohorts need to be interpreted relative to the outcome measure of interest. The outcome variable ‘accuracy’ evaluated final predictions as discrete categories, where predictions could be considered either correct or incorrect. However, the outcome variable ‘AUC’ measured the degree of separability and as previously demonstrated, is a better measure of algorithm performance than accuracy^58,59^.

External validation performance differences between cohorts also may be attributed to intrinsic differences in the number of otoscopic images available for training, ear disease categories, and image capture devices. Cohort A had the lowest internal performance despite having both the greatest number of otoscopic images available for training and number of ear disease categories within overarching ‘abnormal’ classification labels. Although these factors may have contributed to less accurate internal performance due to greater heterogeneity in model learning across each category, these deficiencies were not translated to the model’s ability to generalize compared to Cohort B and C.

Interestingly, models naïve to OME during training were able to achieve an AUC between 0.72 to 0.90 and to differentiate OME from normal TMs in validation cohorts. This achievement may reflect the CNN’s architectural design and the feature extraction layers that were used to extract pertinent features common to normal TMs, ones that are translatable to new cohorts. However, the addition of AOM in Cohort A may have negatively impacted the CNN’s ability to extract pertinent features as, often, AOM, OME, and normal TMs are phenotypically comparable and challenging to differentiate, even among experts^8,9,60,61^.

In a pooled analysis of all three cohorts combined, the DenseNet-161 architecture achieved substantial performance (AUC 0.96) and exceeded the external performance from each individual cohort, respectively. This finding demonstrated that the reduction in performance between internal and external validation, could potentially and in part, be overcome by coming the data into a pooled cohort by sampling a broad collection of otoscopic images from multiple sources.

This study represents the first attempt to evaluate the generalisability of AI-otoscopy algorithms trained and tested on three unique and independently collected databases. Although the image capture devices and data collection protocols varied by each cohort, the deep learning methodology was standardised and uniform between groups. This approach revealed the potential for pre-trained CNNs to extract important features from otoscopic images used during training that could be applied universally to new images. Comparable to a healthcare worker’s evolving knowledge base that improves with otoscopy experience, CNNs demonstrate the potential to identify useful patterns in otoscopic images despite differences in otoscopes, image quality, and underlying ear disease diagnoses. The patterns and features extracted by pre-trained CNNs enable broad differentiation between normal versus abnormal TMs. However, the choice of architecture can impact model performance, with more complex architectures potentially achieving better performance to capture complex features in the otoscopic images. This was found as DenseNet-161, which has the largest number of layers compared to other architectures used, achieved the highest performance to generalise between cohorts. However, more complex architectures may also require more data and computing resources to train and may be more prone to overfitting. As these architectures were pre-trained on large datasets, they can extract features from images that are relevant for many different image classification tasks.

This study has several limitations. First, this study utilises secondary data published online for open-access use. As a result, the data collection protocols, image capture devices, ground-truth definitions, and labelling formats were not standardised across cohorts. Although the models developed for this study evaluated normal versus abnormal classifications, it is plausible that differences in the ear disease definitions and potential misclassifications could influence the models’ performance and overestimate its accuracy. Second, it is unclear if the ear disease diagnoses were based on otoscopic images alone or in concert with tympanometry, audiometry, pneumatic otoscopy, and clinical history. Inter-cohort variability and interpretation bias may exist in the ground-truth labels, potentially contributing to external measurement errors and misrepresenting the algorithm’s generalisability. Third, ground-truth labels were not established by expert consensus. Inter-rater variability and rater bias could impact the overall reliability of ground-truth labels and adversely impact external validation.

The potential for AI-based algorithms to augment otoscopy is promising. Autonomous AI-otoscopy tools have the potential to improve triage and clinical decision-making. In settings where the prevalence of ear disease is high and access to healthcare services is limited (e.g., rural and remote areas; Indigenous communities in North America, Australia, and New Zealand; under-resourced or marginalised populations in sub-Saharan Africa, India, south-east Asia)^12,62,63^, AI-otoscopy algorithms may enhance telehealth programs and empower local healthcare workers to perform otoscopy with more certainty. However, for this to be achieved, AI-otoscopy models must be accurate, reliable, generalizable, accessible in online/offline settings, sustainable, and ethnically constructed and deployed. Early studies have demonstrated that CNNs can achieve substantial performance to interpret otoscopy, although, as shown in this study, external validation performance and generalisability to new settings is limited. Further efforts are required to expand otoscopic image databases to include images from multiple countries, rural versus urban settings, various image capture devices, and include users from various experience levels to account for variability in image capture quality. Establishing standardised diagnostic definitions or consensus from expert panels is required to ensure the ground-truth labels are valid and subsequent models are trained with minimal classification bias. The consideration of data augmentation and pre-processing techniques may be explored to enhance external performance and overcome performance limitations. Otoscopy practices will inevitably vary between clinical settings and a robust, comprehensive, and generalizable AI-otoscopy algorithm is necessary for real-world applications.

## Conclusion

Internal and pooled performance results are comparable to previously reported findings. However, external performance was reduced when applied to new test cohorts. Further efforts are required to explore data augmentation and pre-processing techniques to improve external performance and develop a robust, generalizable algorithm for real-world clinical applications.

## Supplementary Information

Supplementary Information.

## Data Availability

Data that support the findings of this study are available from the corresponding author on reasonable request.
