# Supplementary material for: Evaluating the generalizability of deep learning image classification algorithms to detect middle ear disease using otoscopy
Source: Sci Rep. 2023 Apr 1;13:5368. doi: 10.1038/s41598-023-31921-0 (PMC10067817; doi:10.1038/s41598-023-31921-0)
Supplement: Supplementary file 1 — Supplementary Information. [file 41598_2023_31921_MOESM1_ESM.docx]

Appendix. Table 1. Summary of internal and external performance between Cohorts to differentiate normal versus abnormal otoscopic images trained using ResNet-50, DenseNet-161, VGG16, Vision Transformer, including accuracy, AUC, sensitivity, specificity.

Legend: Acc – accuracy, AUC – area under the curve, Sen – sensitivity, Spec – specificity

| Experiments |  | ResNet-50 |  |  |  |
| --- | --- | --- | --- | --- | --- |
|  |  | Acc | AUC | Sen | Spec |
|  | Internal | 0.78(0.02) | 0.83(0.01) | 0.63(0.03) | 0.85(0.04) |
|  | Chile | 0.75(0.05) | 0.88(0.01) | 0.84(0.08) | 0.71(0.11) |
|  | Ohio | 0.75(0.01) | 0.81(0.02) | 0.88(0.05) | 0.56(0.1) |
|  | Internal | 1.0(0.0) | 1.0(0.0) | 1.0(0.0) | 1.0(0.0) |
|  | Turkey | 0.59(0.04) | 0.62(0.02) | 0.57(0.06) | 0.6(0.08) |
|  | Ohio | 0.76(0.02) | 0.87(0.03) | 0.72(0.1) | 0.82(0.12) |
|  | Internal | 0.92(0.01) | 0.99(0.0) | 0.96(0.02) | 0.86(0.0) |
|  | Turkey | 0.53(0.12) | 0.69(0.01) | 0.66(0.37) | 0.47(0.35) |
|  | Chile | 0.37(0.02) | 0.86(0.01) | 1.0(0.0) | 0.05(0.03) |
|  |  | DenseNet-161 | |  |  |
|  |  | Acc | AUC | Sen | Spec |
|  | Internal | 0.83(0.01) | 0.86(0.01) | 0.71(0.04) | 0.88(0.04) |
|  | Chile | 0.65(0.04) | 0.84(0.03) | 0.93(0.02) | 0.52(0.06) |
|  | Ohio | 0.67(0.03) | 0.71(0.05) | 0.9(0.01) | 0.31(0.08) |
|  | Internal | 1.0(0.0) | 1.0(0.0) | 1.0(0.0) | 1.0(0.0) |
|  | Turkey | 0.56(0.07) | 0.67(0.05) | 0.72(0.21) | 0.48(0.2) |
|  | Ohio | 0.81(0.01) | 0.89(0.01) | 0.81(0.06) | 0.81(0.06) |
|  | Internal | 0.92(0.01) | 0.98(0.0) | 0.94(0.01) | 0.88(0.03) |
|  | Turkey | 0.52(0.04) | 0.66(0.03) | 0.79(0.07) | 0.39(0.09) |
|  | Chile | 0.36(0.01) | 0.92(0.01) | 1.0(0.0) | 0.05(0.01) |
|  |  | VGG16 |  |  |  |
|  |  | Acc | AUC | Sen | Spec |
|  | Internal | 0.82(0.01) | 0.84(0.0) | 0.59(0.03) | 0.92(0.03) |
|  | Chile | 0.8(0.03) | 0.89(0.02) | 0.8(0.04) | 0.8(0.04) |
|  | Ohio | 0.73(0.03) | 0.78(0.05) | 0.82(0.02) | 0.61(0.09) |
|  | Internal | 1.0(0.0) | 1.0(0.0) | 1.0(0.0) | 1.0(0.0) |
|  | Turkey | 0.64(0.02) | 0.63(0.01) | 0.51(0.07) | 0.7(0.05) |
|  | Ohio | 0.77(0.01) | 0.9(0.01) | 0.68(0.03) | 0.91(0.03) |
|  | Internal | 0.94(0.01) | 0.99(0.0) | 0.97(0.01) | 0.9(0.01) |
|  | Turkey | 0.66(0.01) | 0.58(0.02) | 0.34(0.09) | 0.81(0.06) |
|  | Chile | 0.41(0.03) | 0.92(0.02) | 1.0(0.0) | 0.11(0.04) |
|  |  | Vision Transformer | |  |  |
|  |  | Acc | AUC | Sen | Spec |
|  | Internal | 0.82(0.0) | 0.85(0.02) | 0.69(0.04) | 0.89(0.02) |
|  | Chile | 0.62(0.06) | 0.86(0.02) | 0.94(0.02) | 0.47(0.09) |
|  | Ohio | 0.65(0.02) | 0.64(0.06) | 0.91(0.02) | 0.26(0.04) |
|  | Internal | 1.0(0.0) | 1.0(0.0) | 1.0(0.0) | 1.0(0.0) |
|  | Turkey | 0.67(0.03) | 0.62(0.05) | 0.14(0.13) | 0.92(0.1) |
|  | Ohio | 0.67(0.03) | 0.82(0.04) | 0.56(0.06) | 0.85(0.05) |
|  | Internal | 0.96(0.01) | 0.99(0.0) | 0.98(0.01) | 0.93(0.01) |
|  | Turkey | 0.53(0.11) | 0.57(0.03) | 0.61(0.31) | 0.49(0.31) |
